# Patients’ perspectives of telemedicine appointments for psoriatic arthritis during the COVID-19 pandemic: results of a patient-driven pilot survey

**DOI:** 10.1186/s41927-021-00242-y

**Published:** 2022-02-22

**Authors:** Hannah Jethwa, Melanie Brooke, Andrew Parkinson, Emma Dures, Nicola J. Gullick

**Affiliations:** 1 Department of Rheumatology, Hammersmith Hospital, 72 Du Cane Road, London, W12 0HS UK; 2 Bath, UK; 3 Leeds, UK; 4 Academic Rheumatology, Bristol Royal Infirmary and Centre for Healthcare and Clinical Research, University of West of England, Bristol, UK; 5 Department of Rheumatology, University Hospitals Coventry and Warwickshire NHS Trust, Coventry, UK

**Keywords:** Rheumatology, Psoriatic arthritis, Patient perspectives, Telemedicine

## Abstract

**Background:**

Over recent years the lack of patient involvement in the design, set-up and implementation of clinical research studies has been well recognised; as such there has been a drive within research communities to increase patient participation. Patient perspectives on telemedicine differ widely, with variation in whether patients feel remote consultations are beneficial. By means of a patient-driven survey, we aimed to formally evaluate patient perspectives on its benefits and pitfalls, focusing on patients with psoriatic arthritis (PsA).

**Methods:**

An e-survey was developed by two patient representatives on the BritPACT steering committee, with a view to determining unmet needs and the perceived impact on clinical care of virtual consultations amongst patients with PsA.

**Results:**

128 patients responded to the e-survey. 109 patients rated the effectiveness of their telemedicine appointment and, of these, 18% felt their virtual consultation was very/extremely effective compared to an in-clinic consultation and 49% felt it was somewhat/equally as effective; furthermore, 48% (51/107) felt that such virtual consultations would be of benefit to them after the pandemic. 36% of respondents felt their virtual consultation was not as effective as an in-clinic review. Themes identified from open-ended questions included the lack of visual cues, lack of physical examination and effect on rapport and ease of open communication as the main pitfalls of virtual consultations. Patients with well-controlled symptoms appeared more satisfied with remote reviews compared to those with active disease, though on the whole respondents recognised the benefits, such as saving travel time and costs. Those who had an established relationship with their health professional appeared less concerned regarding virtual consultations though a recurring view was that newly diagnosed patients should have in-clinic appointments to build rapport and improve symptom control at an early stage.

**Conclusions:**

Overall patients’ perspectives on virtual consultations varied widely though patients with well-controlled symptoms and those who had a previously established relationship with their healthcare professionals and well-controlled disease appeared more satisfied with remote reviews.

**Supplementary Information:**

The online version contains supplementary material available at 10.1186/s41927-021-00242-y.

## Background

The concept and implementation of telemedicine has been expanding in the USA over the past few decades however in the UK its use had been relatively limited until the Covid-19 pandemic in early 2020 [1]. Telemedicine was rapidly adopted as a method of continuing rheumatology services across the UK, particularly as many patients were advised to shield at home during this period [2]. This dramatic change in healthcare delivery led to significant challenges amongst healthcare management teams, healthcare professionals as well as patients, especially during the early phases of implementation. Over a year later many Rheumatology consultations within the UK continue to be conducted virtually, either by telephone or video-calls, and such remote reviews are likely to continue to some degree for the unforeseeable future. Objective evidence for the effectiveness of virtual consultations is limited and, until recently, qualitative studies of telemedicine amongst Rheumatology departments predominantly involved patients with Rheumatoid arthritis, with limited data on healthcare professional and patient perspectives on its delivery and outcomes amongst those with other Rheumatological diseases [3].

Over recent years the lack of patient involvement in the design, set-up and implementation of clinical research studies has been well-recognised. As such there has been a drive within research communities to increase patient participation and many committees. The British Psoriatic Arthritis Consortium (BritPACT), have recruited patient partners to try to aid with this. MB and AP are both patient representatives on the British Psoriatic Arthritis Consortium (BritPACT) steering committee. Their roles within the organisation reflect views, insights and lived experience of patients with psoriatic arthritis (PsA). MB also works in patient engagement, peer support, is actively engaged with a wide pool of patients and has some previous experience of designing and reviewing e-surveys. AP has shared his lived experience with researchers on projects focussed on psoriatic arthritis for a number of years.

The aim of this study was to evaluate patient perspectives on the use of telemedicine amongst individuals with psoriatic arthritis, with a view to determining unmet needs and the perceived impact on clinical care by means of a patient-driven survey. The survey was designed by patients, for patients, to share experiences of virtual care of psoriatic arthritis during the Covid-19 pandemic. We present the results of this patient-driven survey.

## Methods

MB and AP developed an electronic survey with 18 questions covering demographics, treatment details, as well as type and effectiveness of telemedicine appointments (supplementary data 1). The survey was designed from a patient-to-patient discussion perspective with a broad range of questions rather than only as an exploratory clinical tool. The survey was reviewed by four additional BritPACT patient partners as well as three clinical expert members of the BritPACT steering committee prior to final approval. Each question provided options to select an answer and many gave opportunity for free text responses in order to give the participants a wider opportunity to express their experiences.

The survey was distributed by the end of January 2021 via the British Psoriatic Arthritis Consortium (BritPACT) patient network, as well as through other patient organisations and support groups (Psoriasis and Psoriatic Arthritis Alliance (PAPAA), Psoriasis Association, Bath Institute of Rheumatic Diseases, Patient Arthritis Support Group (PsAZZ and PsA HQ) and shared via social media.

Results were collated after approximately 6 weeks following survey distribution. In addition to tick-box questions, patients were also invited to respond to open-ended questions on their experience with telemedicine. Open-ended responses ranged from a single word to paragraphs of around 100 words, with most being one sentence long. ED analysed these responses using a quasi-qualitative manifest content analysis [4].This process involved collating the textual data and grouping responses that reflected a shared meaning. These grouped responses were then synthesised, summarised and presented as categories. This was done for the whole survey, rather than per question, because there was overlap in the content of patients’ open-ended responses.

## Results

128 patients responded to the e-survey, 14 of whom reported to not having had a virtual rheumatology consultation, the vast majority of which were conducted via telephone. Most respondents (76%) identified as female and the majority were between 45 and 74 years of age (77%). Most telemedicine appointments had occurred with a rheumatologist or a rheumatology nurse, although virtual physiotherapy, occupational therapy and GP appointments also took place, the latter of which were all in relation to psoriatic arthritis.

Waiting times on the day of appointment were rated as better by 41/108 patients (38%) or about the same by 60/108 (56%). Only 7/108 (6%) rated waiting times as worse in-clinic appointments.

**Fig. 1:**
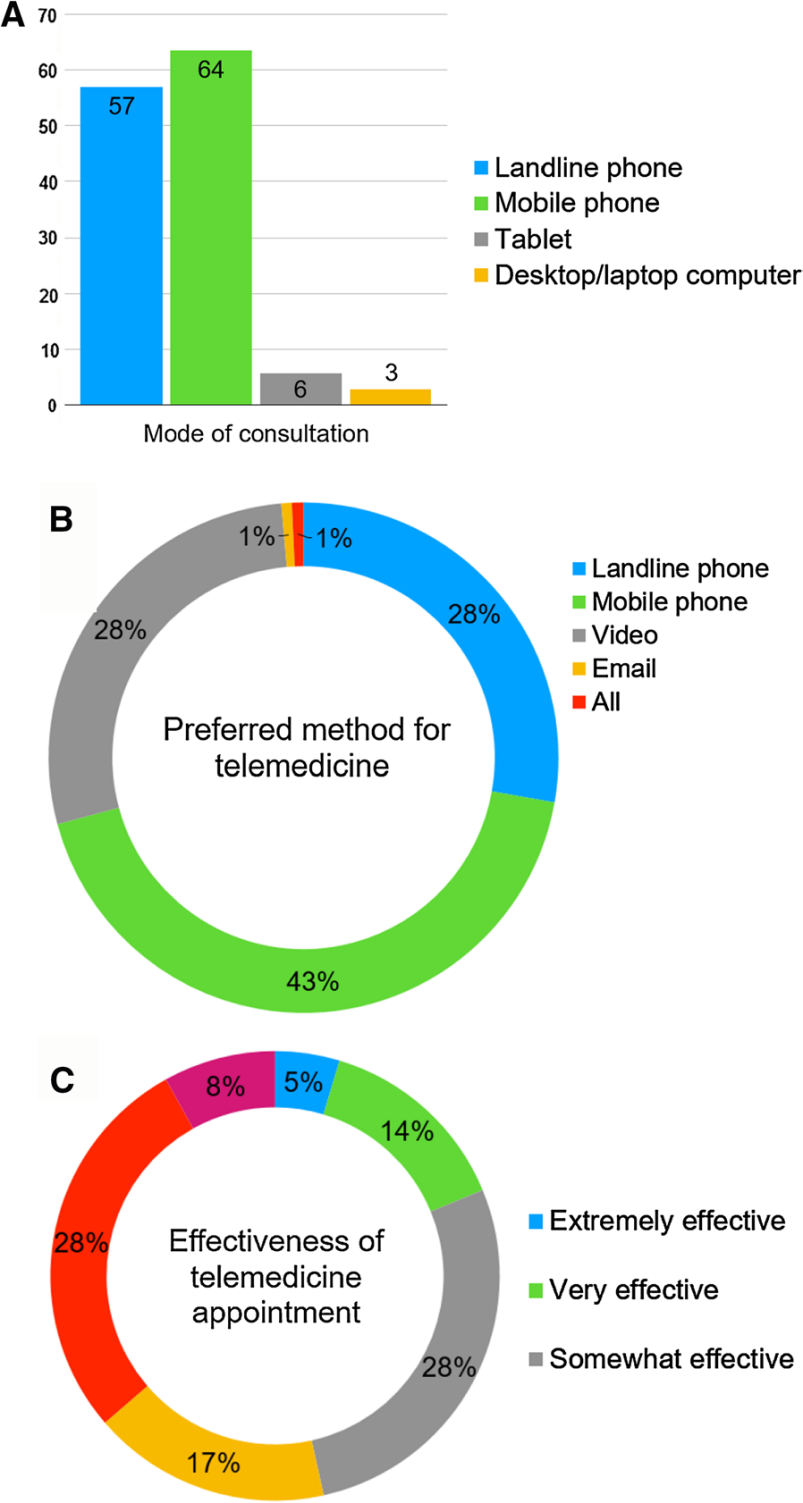
Patient perspectives of telemedicine. **a** mode of consultation, **b** preferred method for consultation, **c** effectiveness of appointment. Patients were asked how their virtual consultations had taken place, what their preferred method of communication for a virtual appointment would be and how effective their telemedicine appointment had been

109 patients rated the effectiveness of their telemedicine appointment: 60 patients (63%) found their appointments to be either equally as or more effective compared to in-clinic consultations, although 31 patients (28%) felt this method of assessment was not as effective and 9 patients (8%) felt it was not at all effective (Fig. 1) (Additional file 1).

Almost half of patients 51/107 (48%) felt that telemedicine appointments would be of benefit to them after the pandemic; 28 (26%) patients did not feel that telemedicine appointments would benefit them and 28 (26%) were unsure. 53/107 (50%) of patients felt that they could be more open about their PsA at an in-clinic appointment. However, 6 patients felt they could be more open in a telemedicine appointment; the remainder did not feel there was a difference.

The majority of patients did not feel that continued remote consultations would impact on the ability to achieve remission of their condition (68/106, 62%) or ability to take part in research studies (67/105, 64%). 58/105 (55%) patients, however, felt that ongoing remote consultations would not provide holistic care and 57/105 (54%) felt that remote consultations would not allow as good rapport to be built with their clinical team compared to in-clinic reviews.

Five categories were identified to present patients’ views on telemedicine appointments:

1. Rheumatology requires physical assessments

*‘In Rheumatology so much is about being hands on and assessing the joints’*

Respondents stated the importance of rheumatology health professionals being able to see and touch patients’ joints and skin as part of providing care. This was not only about joints, but also about non-verbal cues and body language and being able to see the whole person and asses how they are coping, including their mood and overall wellbeing.

(2) Routine check-ups versus complex consultations

*‘It would be fine for ‘stable’ patients but* in-clinic *appointments need to be available for people whose symptoms are not stable’*

Respondents whose symptoms were stable were more positive about receiving optimal care via telemedicine and many reported that video or telephone calls would be fine for monitoring appointments. However, there were concerns about the potential for patients’ health to deteriorate if they are not seen in person over a long period. Respondents also cited examples of rheumatology health professionals identifying issues in person that patients would not have reported on a call.

(3) A flexible and responsive system

*‘A hybrid of telemedicine and in person would be good’*

Respondents recognised potential benefits of telemedicine at both an individual level (saving on travel time and costs) and system level (possible resource savings for the NHS). However, they explained that it would be important that they could switch from a telemedicine to an in-clinic appointment if needed, and at short notice. A telemedicine appointment should not delay an in-person appointment.

(4) Pre-existing trust and rapport with the rheumatology team

*‘I feel my direct contact with the team has built up valuable trust and understanding of my difficulties as a patient’*

Respondents were often less concerned about telemedicine if they had established relationships with the health professional that they were talking to. A recurring view was that patients newly diagnosed should have in-clinic appointments to build rapport and get control of their symptoms. There was also concern about care provision for patients who might struggle with telemedicine, such as people who do not have English as a first language and *‘the ability of the doctor or nurse to understand, translate and express themselves clearly’*. A perception among some respondents was that consultants have more medical expertise and can prescribe/make changes to treatment, while nurses were more emphatic and better at communicating in lay terms.

(5) Telemedicine is here to stay

‘New way of working but I can adapt and I'm sure professionals can as well’

At this point, patients providing free text responses often expressed a preference for video calls over telephone calls and recognised that the rapid move to telemedicine was a work in progress. For some, telemedicine was working well, and they were highly satisfied with their remote consultations. For some, it was less satisfactory, due to the quality of the communication and the lack of physical examination. In the longer-term, some patients thought that telemedicine was likely to become part of their care and that provision might improve as it becomes normalised, for example the quality of the video and/or telephone calls. Some tips for better versions of telemedicine include sending text reminders and use of a pre-appointment check list.

## Discussion

The COVID-19 pandemic has dramatically changed the way routine, outpatient healthcare is provided, with the majority of consultations being conducted virtually during the peak of the pandemic as well as to date. It is likely that telemedicine is going to remain as standard practice from now onwards where appropriate and, as such, research is required to identify its efficacy compared an in-clinic consultations, cost considerations as well as the optimal technological modalities required for its long-term implementation. Although virtual consultations were a requirement during the peak of the pandemic to minimise infection risk for patients, many potential long-term benefits exist, such as improved convenience for patients with reduced travel time and time off work, and potentially reduced costs to healthcare providers [5, 6]. Some disadvantages, however, include potential difficulties building rapport between patients and healthcare providers as well as difficulty with accurate clinical assessments in patients with active disease. For the latter, video-consultations are preferable over telephone consultations though the possibilities for examination remains limited.

Most studies on the utility of telemedicine in rheumatology have surveyed rheumatologists rather than patients. To date, a small number of patient perspective studies on the use of telemedicine amongst patients with rheumatological disorders include predominantly patients with rheumatoid arthritis [7]. We report the widespread use of telemedicine by patients with PsA during the COVID-19 pandemic across various centres in the UK, with the majority of virtual consultations taking place via telephone.

Although patients had reported the preferred method of contact as telephone, free text responses suggested that video appointments could allow better assessment of symptoms. Although the different aspects which constitute an ‘effective’ consultation are broad and likely variable between individuals, on the whole patients felt that if their disease was well-controlled virtual consultations were an adequate modality of clinical assessment. Patient satisfaction was situation dependent, and patients noted a preference for a physical examination, especially if disease was active. A larger survey, which also recruited predominantly patients with RA from North America also noted higher satisfaction with a virtual appointment if well controlled disease, with lower satisfaction if mild or moderate disease. Preference for video consultations was also mixed, with 43% expressing a preference for video appointments, while 43% had no preference [7]. A further study from Poland noted that telemedicine (predominantly by telephone) expressed doubts over the value of telemedicine, reporting lack of examination, concerns about being able to explain symptoms and lack of ability to perform additional tests such as blood tests, which would ordinarily take place at the time of an in-clinic appointment. However, patients valued certain aspects of a traditional consultation which can be reproduced during a teleconsultation such as direct conversation with a doctor and communication of results [8].

There is likely to be some disparity between patient perspectives depending on their underlying disease. For example, studies in patients with systemic lupus erythematosus (SLE) have identified a more negative impact of virtual consultations compared to our survey, although objectives and study design differed [9].

Finally, the experience of patients designing and leading the survey project has been valuable, but not without challenges. MB and AP designed the survey questions, with contributions of BritPACT patient partners. Although both had experience of contributing to research and reviewing projects as patient participants or reviewers, neither had planned or led projects in their entirety, and acknowledge support from multiple people and organisations in design, distribution, and academic writing, and have gained new skills during the project.

In addition to giving insight of patients’ perspectives on the use of virtual consultations amongst those with PsA, this ‘patient driven e-survey’ raises the question of whether the survey design and implementation ‘by patients for patients’ allows for freer and more honest expression from respondents.

Some limitations of the survey include that clinical data on duration of disease was not identified and, as such, there may be some discrepancy in patient satisfaction of virtual consultations in newly diagnosed patients compared to those in whom treatment was already long-established. The latter group are more likely to have developed a rapport with their clinical team and therefore may feel more confident with their consultations being conducted virtually.

Furthermore, the survey was conducted electronically and shared by patient organisations and via social media. It is possible that responses are not fully representative of the UK population of patients with PsA, as patients with limited or no access to technology may have different views of telemedicine compared to those who are regular users of technologies. Additionally, the survey was only conducted in English and those with language- or perceived educational-barriers may not have participated and, due to the nature of survey distribution, we are unable to ascertain response rates compared to the number of patients who had access to the survey itself. As such, it is difficult to speculate whether patient-driven surveys are more likely to have higher response rates compared to surveys developed and distributed by healthcare professionals.

Due to the nature of the e-survey it was not possible to determine overall response rates and whether there were systematic differences in those who did/did not respond. Furthermore the survey was predominantly distributed to patients who are part of PsA networks or patient organisations and these patients may be more highly motivated with greater self-efficacy and involvement in their care; as such it may not accurately reflect views of the wider patient population. A wider survey taking these factors into account may yield different results and repeating a similar survey post-pandemic may be helpful to determine whether current perspectives on virtual consultations were affected by the necessity of this modality due to governmental restrictions rather than patient preference alone.

Patient-driven surveys may allow for more open responses from patients and questions written by patients may be better and more relevant in some topic areas as they are aware of the issues patients face first-hand; qualitative and quantitative research is required to review whether this is the case and increased experience of patient-driven surveys may enhance both aspects.

## Conclusions

Although there are many benefits to virtual consultations, limitations according to patients with active symptoms of disease include the limited ability for a physical examination. As such, although many patients are aware telemedicine is likely to be the ‘new norm’ to some extent indefinitely, a hybrid model of virtual as well as in-clinic reviews, with the possibility of seeing a healthcare professional in-person in the case of a flare, is the most preferable model of healthcare delivery.

Patient perspectives on service design and changes are increasingly important. More engagement of patient partners should be sought after by clinical departments in order to improve our understanding as this, in turn, may help improve the patient satisfaction aspect of clinical care outcomes.

## Supplementary Information

**Additional file 1**: Summary of results from e-survey.

**Additional file 2**: Lay summary.

## Data Availability

The datasets used and analysed during the current study are available from the corresponding author on reasonable request.
