# Supplementary material for: Patients’ perspectives of telemedicine appointments for psoriatic arthritis during the COVID-19 pandemic: results of a patient-driven pilot survey
Source: BMC Rheumatol. 2022 Feb 22;6:13. doi: 10.1186/s41927-021-00242-y (PMC8860501; doi:10.1186/s41927-021-00242-y)
Supplement: Supplementary file 1 — Additional file 1: Summary of results from e-survey. [file 41927_2021_242_MOESM1_ESM.pdf]

Q1 Please tick one of the following choices - are you:

Answered: 128 Skipped: 1

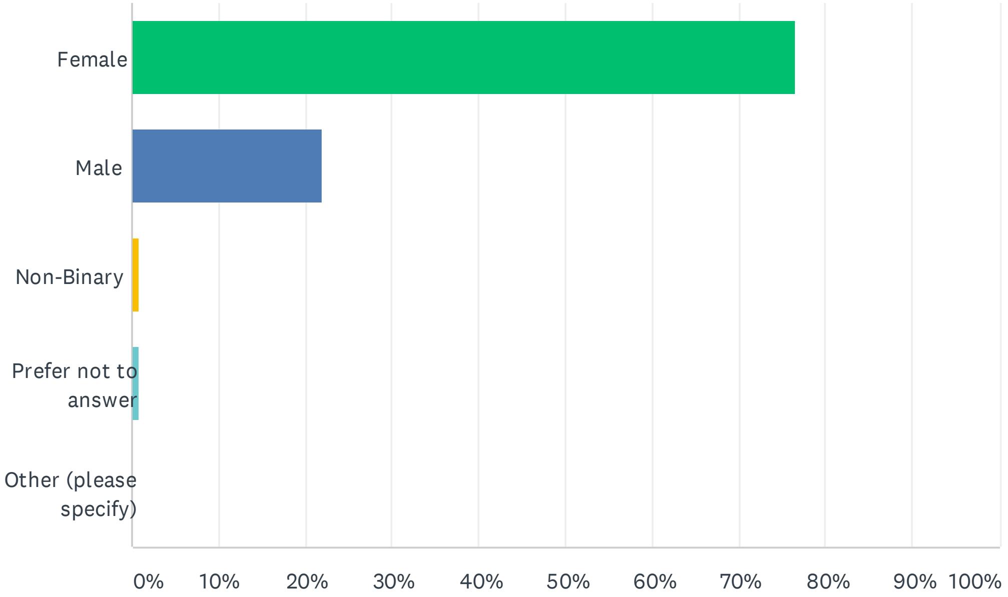

ANSWER CHOICES	RESPONSES	
Female	76.56%	98
Male	21.88%	28
Non-Binary	0.78%	1
Prefer not to answer	0.78%	1
Other (please specify)	0.00%	0
TOTAL		128

#	OTHER (PLEASE SPECIFY)	DATE
	There are no responses.	

Q2 What is your age?

Answered: 128 Skipped: 1

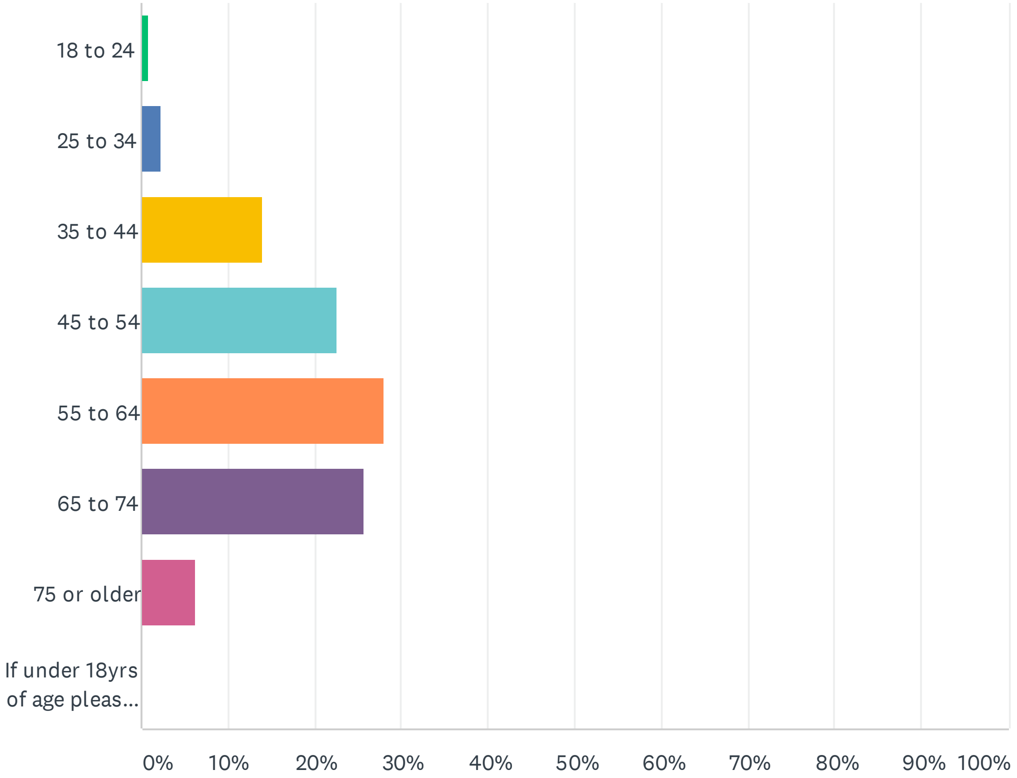

ANSWER CHOICES	RESPONSES	
18 to 24	0.78%	1
25 to 34	2.34%	3
35 to 44	14.06%	18
45 to 54	22.66%	29
55 to 64	28.13%	36
65 to 74	25.78%	33
75 or older	6.25%	8
If under 18yrs of age please specify:	0.00%	0
TOTAL		128

#	IF UNDER 18YRS OF AGE PLEASE SPECIFY:	DATE
	There are no responses.	

Q3 Please tell us about the medications you take for your Psoriatic Arthritis (tick all that apply)

Answered: 126 Skipped: 3

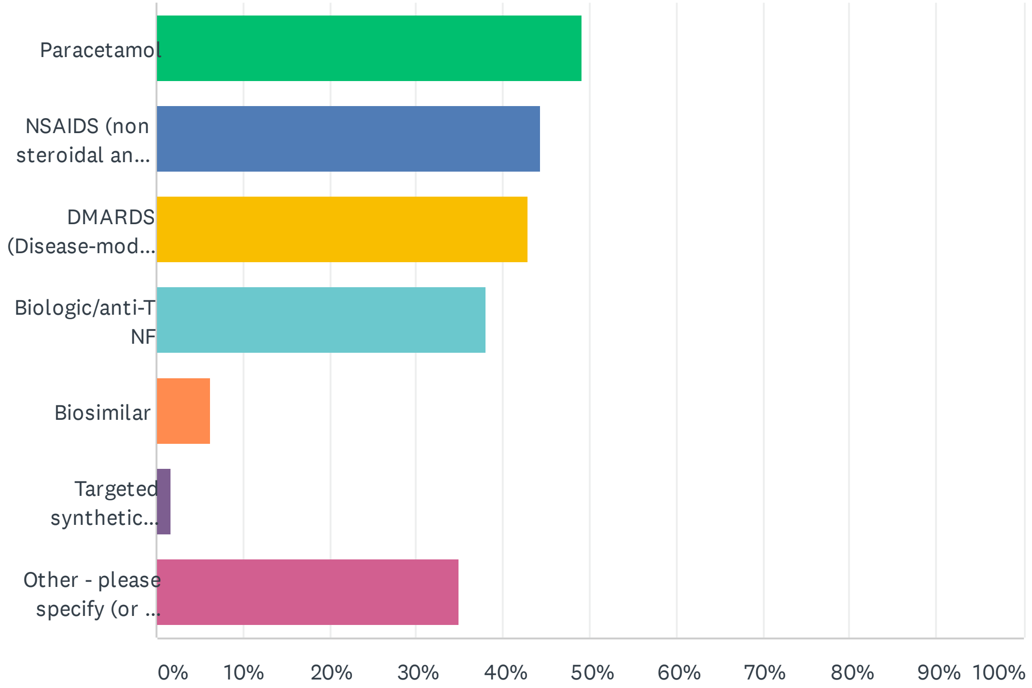

ANSWER CHOICES	RESPONSES	
Paracetamol	49.21%	62
NSAIDS (non steroidal anti inflammatory drugs)	44.44%	56
DMARDS (Disease-modifying antirheumatic drugs)	42.86%	54
Biologic/anti-TNF	38.10%	48
Biosimilar	6.35%	8
Targeted synthetic tablet	1.59%	2
Other - please specify (or if not sure what type of med you take just enter the medication name from the packet below)	34.92%	44
Total Respondents: 126		

#	OTHER - PLEASE SPECIFY (OR IF NOT SURE WHAT TYPE OF MED YOU TAKE JUST ENTER THE MEDICATION NAME FROM THE PACKET BELOW)	DATE
1	Once a while Advil . Daily .25 Dexamethasole After breakfast with other regular medicines for uaric acids and hart related	3/9/2021 8:00 AM
2	Medicinal Cannabis	3/8/2021 10:39 AM
3	Tramadol and amitriptyline	3/1/2021 3:16 PM
4	Co-codamol 15/500	2/27/2021 10:12 PM
5	Antimetabolite - Methotrexate	2/24/2021 10:46 AM
6	Ortezla	2/24/2021 12:21 AM
7	Steroid injection	2/17/2021 8:45 PM
8	pain killers... caflam drug pill name ... voren also pill name ...	2/15/2021 5:04 PM
9	Dihydrocodeine	2/5/2021 7:31 AM
10	Methotrexate and Leflunomide	2/4/2021 6:26 PM
11	Codeine	2/4/2021 3:04 PM
12	Methotrexate	2/4/2021 2:59 PM
13	I have chosen not to take medication	2/4/2021 2:54 PM
14	Sulphasalazine	2/4/2021 1:47 PM
15	Get femoston conti	2/4/2021 11:54 AM
16	Apremilast	2/4/2021 11:52 AM
17	Codeine, amitryptiline	2/3/2021 4:22 PM
18	Amitryptiline Boswellia (not prescription)	2/3/2021 1:03 PM
19	Butec patches 10mcgs/hr, diclofenac gel,	2/1/2021 9:42 PM
20	Vitamin D, Flax Seed Oil, curcumin capsules with pepper	2/1/2021 1:35 PM
21	.	1/31/2021 9:43 PM
22	Etericoxib Leflunomide Ustekinimab	1/31/2021 1:25 PM
23	Homeopathic remedies	1/31/2021 11:02 AM
24	Methotrexate, Omeprazole	1/30/2021 6:19 PM
25	Stopped MTX after 4 mths due to effects on liver!	1/29/2021 11:42 PM
26	Steroid injections	1/29/2021 9:45 PM
27	Methotrexate	1/29/2021 7:54 PM
28	Cocodomol	1/29/2021 5:48 PM
29	Prednisolone	1/29/2021 4:20 PM
30	Amitriptyline	1/29/2021 3:48 PM
31	Hydroxychloroquine	1/29/2021 3:46 PM
32	Folic acid	1/29/2021 3:23 PM
33	Methotrexate (one metojet pen per week)	1/29/2021 3:07 PM
34	Methotrexate Sulphasalazine	1/29/2021 2:54 PM
35	Diazepam and tramadol	1/29/2021 2:36 PM
36	Leflunomide and Methotrexate	1/29/2021 2:33 PM
37	Would be on NSAIDS but they are contraindicated for me for other health reasons	1/29/2021 2:31 PM
38	Anticonvulsants	1/29/2021 2:28 PM
39	Duloxotine, Gabapentin, Co-codamol, tramadol, methotrexate, Humira injections, folic acid,	1/29/2021 2:21 PM

	omeprozale	
40	co-codamol	1/29/2021 2:11 PM
41	Naproxin	1/29/2021 2:08 PM
42	Steroids	1/29/2021 2:07 PM
43	Benepali - not sure if I should tick Biological/antiTNF or Biosimilar	1/29/2021 1:56 PM
44	Sulfasalizine	1/27/2021 6:06 PM

Q4 Have you had a telemedicine appointment during 2020/2021?

Answered: 112 Skipped: 17

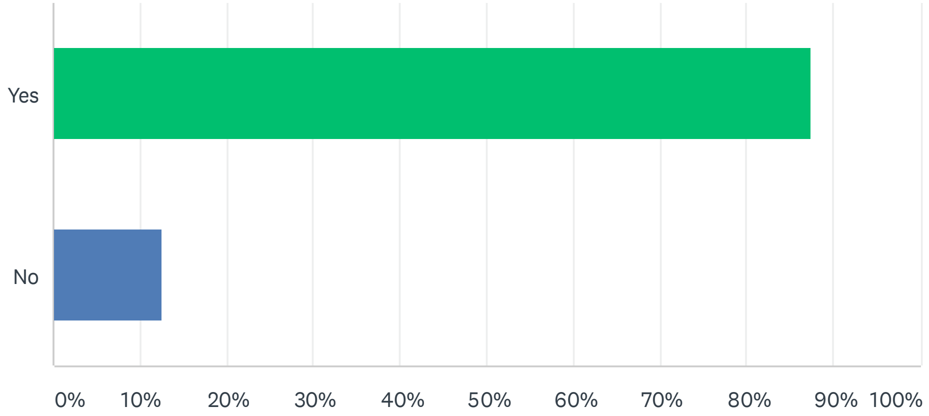

ANSWER CHOICES	RESPONSES	
Yes	87.50%	98
No	12.50%	14
TOTAL		112

Q5 What means of telemedicine have you used for a rheumatology appointment? Please tick all that apply

Answered: 110 Skipped: 19

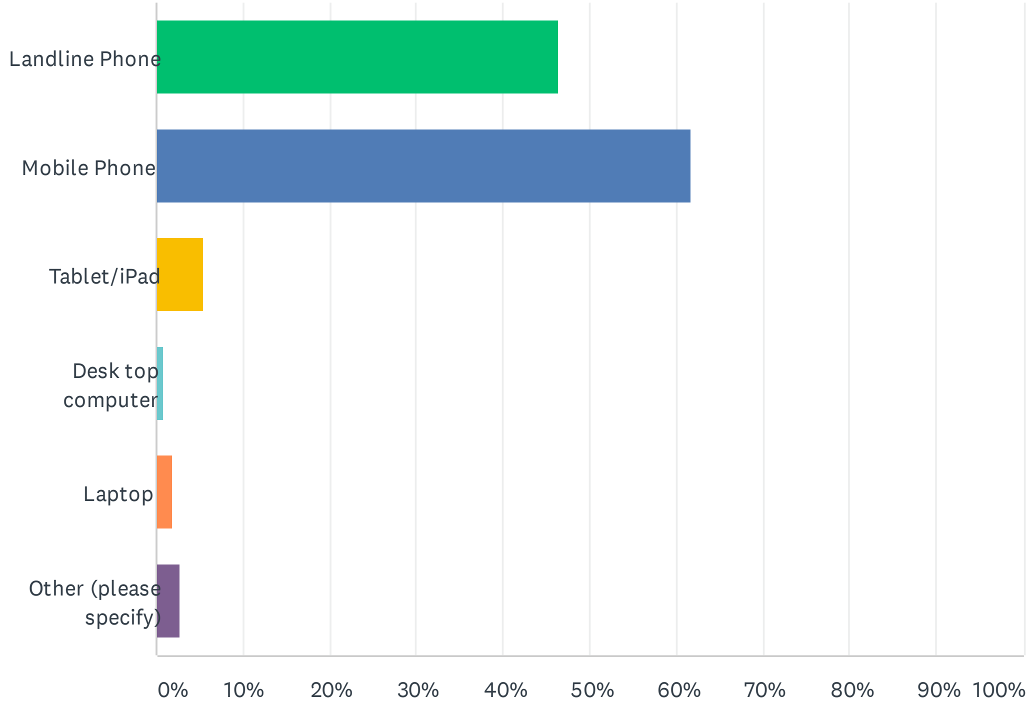

ANSWER CHOICES	RESPONSES	
Landline Phone	46.36%	51
Mobile Phone	61.82%	68
Tablet/iPad	5.45%	6
Desk top computer	0.91%	1
Laptop	1.82%	2
Other (please specify)	2.73%	3
Total Respondents: 110		

#	OTHER (PLEASE SPECIFY)	DATE
1	not any one at all ...	2/15/2021 5:09 PM
2	Not applicable	2/4/2021 3:08 PM
3	No appointments	1/29/2021 3:47 PM

Q6 What is your preferred means of telemedicine? Please tick all that apply

Answered: 111 Skipped: 18

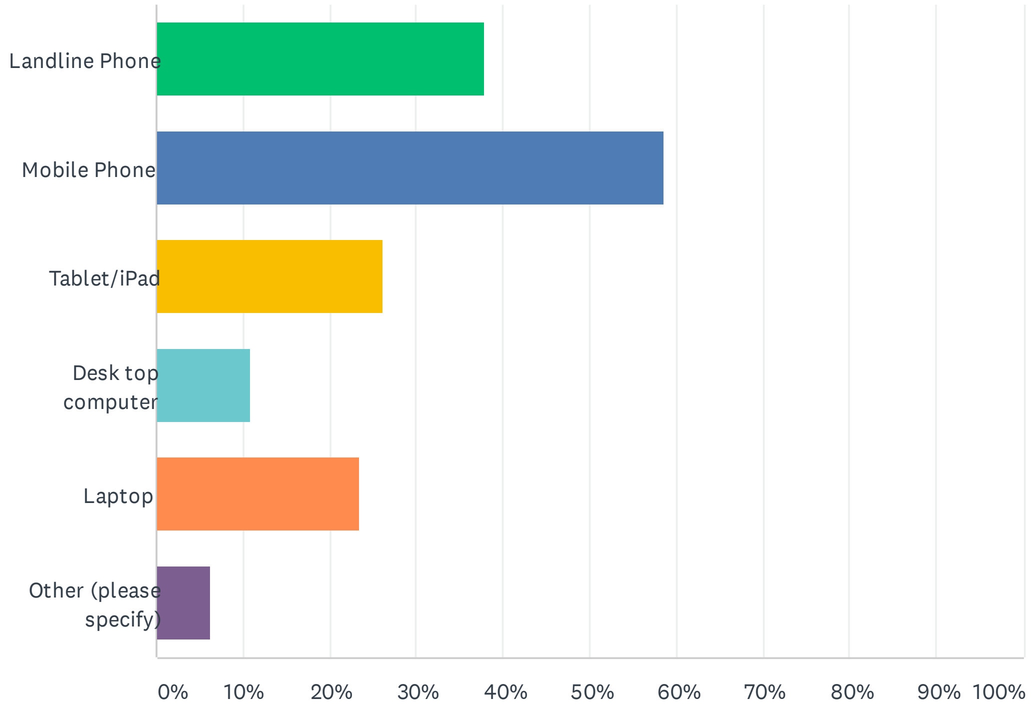

ANSWER CHOICES	RESPONSES	
Landline Phone	37.84%	42
Mobile Phone	58.56%	65
Tablet/iPad	26.13%	29
Desk top computer	10.81%	12
Laptop	23.42%	26
Other (please specify)	6.31%	7
Total Respondents: 111		

#	OTHER (PLEASE SPECIFY)	DATE
1	get connected by all means of communication ...	2/15/2021 5:09 PM
2	Do NOT want telemedicine, need a proper physical examination.	2/4/2021 3:08 PM
3	Email	2/4/2021 3:03 PM
4	I am happy to use any phone, laptop or tablet	2/4/2021 12:59 PM
5	Any method that would allow video rather than just audio would be my preference	2/3/2021 1:05 PM
6	I would prefer to see the persons video call atm	2/1/2021 9:44 PM
7	Zoom - so you can see people's faces	1/29/2021 4:23 PM

Q7 Which health care professional(s) have you had telemedicine calls with? Please tick all that apply

Answered: 106 Skipped: 23

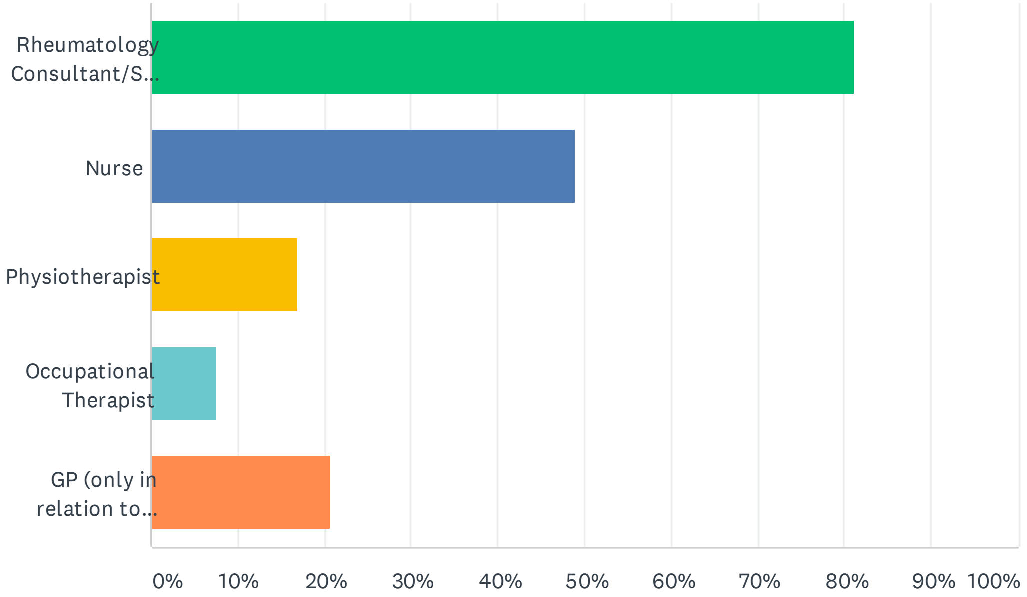

ANSWER CHOICES	RESPONSES	
Rheumatology Consultant/Specialist	81.13%	86
Nurse	49.06%	52
Physiotherapist	16.98%	18
Occupational Therapist	7.55%	8
GP (only in relation to your rheumatic condition for this survey)	20.75%	22
Total Respondents: 106		

#	OTHER (PLEASE SPECIFY)	DATE
1	Never take . Once take appointment with Dr of Rumatology And he detect me as psoriatic Arthyritis to me . I am better with my experiment and controlling .	3/9/2021 8:06 AM
2	Orthopaedic Consultant	3/1/2021 3:19 PM
3	Hospital Pharmacist	2/16/2021 11:33 AM
4	non as mentioned above …	2/15/2021 5:09 PM
5	Dermatology Consultants	2/5/2021 7:34 AM
6	Not applicable	2/4/2021 3:08 PM
7	Dermatologist	2/4/2021 1:49 PM
8	Dermatology	1/31/2021 9:45 PM
9	None	1/29/2021 3:47 PM
10	Dermatologist	1/29/2021 2:14 PM
11	Dermatologist	1/27/2021 6:09 PM

Q8 How was the 'waiting time' of your telemedicine appointment versus the time you were told to expect a call and compared to a face to face clinic appointment? Please pick one answer:

Answered: 108 Skipped: 21

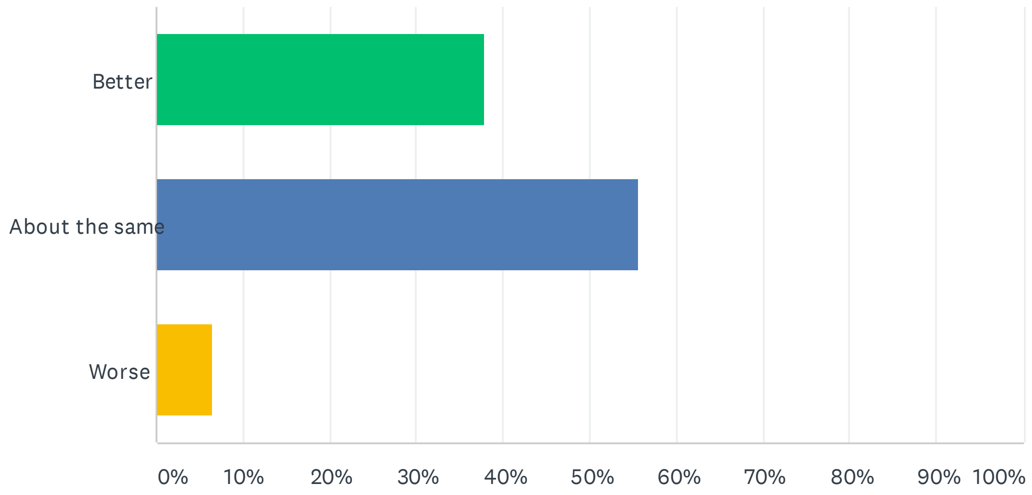

ANSWER CHOICES	RESPONSES	
Better	37.96%	41
About the same	55.56%	60
Worse	6.48%	7
TOTAL		108

Q9 How effective do you feel your telemedicine appointment was (compared to a face to face clinic appointment)? Please pick one answer:

Answered: 109 Skipped: 20

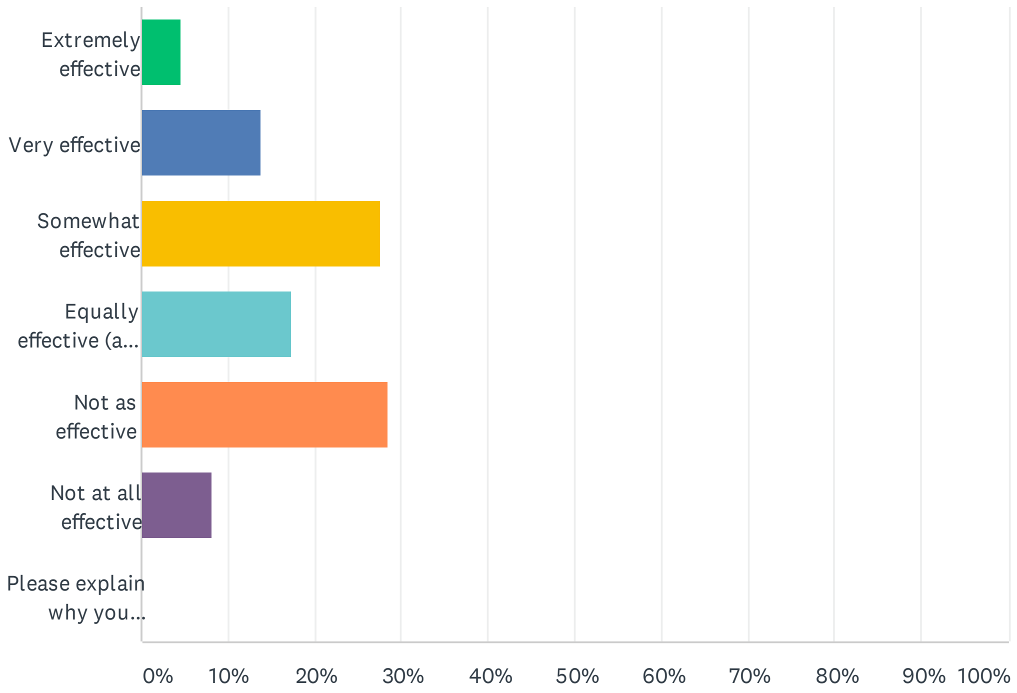

ANSWER CHOICES	RESPONSES	
Extremely effective	4.59%	5
Very effective	13.76%	15
Somewhat effective	27.52%	30
Equally effective (as a regular appointment)	17.43%	19
Not as effective	28.44%	31
Not at all effective	8.26%	9
Please explain why you liked/disliked your telemedicine appointment?	0.00%	0
TOTAL		109

#	PLEASE EXPLAIN WHY YOU LIKED/DISLIKED YOUR TELEMEDICINE APPOINTMENT?	DATE
	There are no responses.	

Q10 In your own words, is there anything that could have been done differently to improve your telemedicine appointment?

Answered: 91 Skipped: 38

#	RESPONSES	DATE
1	Consider My views and Experiment .	3/9/2021 8:06 AM
2	I could have written down more questions and queries I had but forgot.	3/3/2021 9:25 AM
3	Face time not over the phone as that way you can show where it hurts, or any visual problems tat you can not see when on the phone	3/1/2021 3:19 PM
4	Would have preferred video call rather than phone call	2/27/2021 10:14 PM
5	A virtual face-to-face online might have been better - but a telephone call was fine - because it was a regular catch-up appointment and my arthritis is well-controlled on current treatment and I've had no new problems to talk about (other than my concerns about taking methotrexate would affect my susceptibility/immune response to COVID-19).	2/27/2021 4:47 PM
6	Missing any visual observation, so some video element might have helped	2/27/2021 9:08 AM
7	Not really. Nothing replaces face to face appointments.	2/23/2021 6:08 PM
8	I would have liked a text day before just as a reminder	2/19/2021 8:05 PM
9	The proposed video cal didn't work, so my consultant J St rang me.	2/18/2021 7:21 PM
10	No my consultant was very good	2/17/2021 8:46 PM
11	Visual contact so body language can be seen.	2/16/2021 11:33 AM
12	Not sure	2/16/2021 10:56 AM
13	It would have been better to talk on the landline rather than my mobile (bad reception), but I did appreciate being able to talk to my consultant.	2/15/2021 10:48 PM
14	i don't know ... i never had such experience ...	2/15/2021 5:09 PM
15	Use better phones was hard to hear	2/15/2021 4:29 PM
16	Not sure but would have liked a FaceTime call	2/12/2021 10:21 PM
17	A video call rather than telephone would be better	2/7/2021 3:01 PM
18	Rather than a phone call a face to face on zoom or something would be better	2/6/2021 11:13 PM
19	email me a copy of the medic's notes, instead of posting it, to save money on paper & postage	2/5/2021 7:34 AM
20	I felt a bit rushed but I know they are very busy.	2/4/2021 6:28 PM
21	One by phone was (with registrar) was poor and essentially pointless, later one by video call (with consultant) was good and worthwhile, video every time is my preference.	2/4/2021 4:48 PM
22	No regular blood test over the year to monitor methotrexate side effects or monitor inflammation activity. Also waiting for over a year for surgery to arthritic wrist.	2/4/2021 4:11 PM
23	No - it was good and effective.	2/4/2021 3:17 PM
24	Yes I feel a zoom call would have been better as she could have visually seen my knee . Felt she didnt hear what I was trying to say / describe.	2/4/2021 3:15 PM
25	Scrap the idea of this type of consultation. Proper appointments with physical examination is required.	2/4/2021 3:08 PM
26	I just think face to face is much better, it’s easier to explain problems, show physical conditions and changes that can’t be adequately described on the phone. It’s more personal than the phone and a relationship with your consultant is better when you meet in person, trust develops and issues better talked about. Often get tongue tied on the phone and forget what needs to be said. Feels more rushed.	2/4/2021 3:03 PM
27	Longer appointment	2/4/2021 3:00 PM
28	Without examining joints where I have pain how can a diagnosis be made.	2/4/2021 3:00 PM
29	Because examinations art difficult to perform on TeleMed there are disadvantages, but overall my experience is most beneficial.	2/4/2021 2:57 PM
30	perhaps a video call?	2/4/2021 2:49 PM
31	Better and more reliable internet speed to allow internet access	2/4/2021 2:46 PM

32	A Zoom call might have been better as you can see the other persons face.	2/4/2021 2:35 PM
33	I didn’t feel that the person I spoke to was particularly interested in my condition or doing anything about anything. I feel it was mostly a formality. I only had one contact and that was last April. Normally I would be seen every 6 months	2/4/2021 2:26 PM
34	To make video calls easier	2/4/2021 2:15 PM
35	Doctor to have time to go through my file before the call	2/4/2021 2:12 PM
36	I’m not sure. I suppose I felt a bit hurried.	2/4/2021 1:49 PM
37	Visual examination of joints via video link	2/4/2021 1:34 PM
38	No	2/4/2021 12:59 PM
39	Would have preferred face to face as could not describe where pain was properly	2/4/2021 12:57 PM
40	I think it might have been good to have it as a Video call rather than a phone call. With Psoriatic Arthritis (and all forms i guess) having someone see you is the aim of the appointments. Not just to look at joints and assess skin etc, but also to look at the person to see how they're coping. Some people might not be as open and honest as I have been on my phone call appointments and might just bottle up other things which might be contributing to their pain/flare/fatigue etc. You can tell a lot from looking at a person's face and how they respond to "how are you doing at the moment" .. is it a genuine "I am doing fine".	2/4/2021 12:37 PM
41	Not really but I was discharged following my telephone call but this was not mentioned during the call so I found out in the letter following the call! Most disappointing	2/4/2021 12:21 PM
42	No	2/4/2021 12:14 PM
43	It's extremely difficult to do any kind of physical assessment via telephone, even with videocall capacity	2/4/2021 12:14 PM
44	No only I could've added more things to my list I wanted to speak about	2/4/2021 11:55 AM
45	No	2/3/2021 4:48 PM
46	Excellent and very thorough call but obviously can't examine or see joints	2/3/2021 4:23 PM
47	My experience was a good one, having met my constant many times made it easier.	2/3/2021 2:04 PM
48	To have video as well as audio	2/3/2021 1:05 PM
49	No best appointments I’ve ever had	2/3/2021 12:50 PM
50	Had a list of things I wanted to ask.	2/3/2021 12:11 PM
51	I would have preferred a video facility only in terms of it feeling a more normal consultation	2/2/2021 9:30 AM
52	A video rather than just telephone, a more structured appointment, questions could be sent so you know what to say etc	2/1/2021 9:44 PM
53	No, they did their best	2/1/2021 3:05 PM
54	I don’t think so - in Rheumatology so much is about being hands on and assessing the joints. This could not be done. Also the visual feedback and non verbal cues get missed in phone consults (I am a GP myself)	2/1/2021 1:38 PM
55	Being able to see the doctor/nurse eg Zoom, WhatsApp	2/1/2021 1:03 PM
56	Video would be an improvement. Facial gesture is as important as voice inflexion as carrier of clues for diagnosis	2/1/2021 12:37 PM
57	My appointment with the nurse would have been better by video My physio appointments were video and really good	1/31/2021 1:28 PM
58	No physical examination possible, important when assessing joint pain	1/31/2021 11:04 AM
59	I think it was fine given the circumstances.	1/30/2021 6:20 PM
60	Had a video call so the consultant could see my joints/condition	1/29/2021 10:24 PM
61	It was fine. If I felt I needed face to face appointment I know I can request it in the phone appointment.	1/29/2021 9:48 PM
62	Not sure. I missed the direct personal assessment of my condition.	1/29/2021 7:56 PM

63	Not certain how it could be Improved	1/29/2021 6:06 PM
64	Video call as joints needed checking visual would be better	1/29/2021 5:49 PM
65	No	1/29/2021 5:37 PM
66	Video call would be better and nearer to face to face	1/29/2021 5:03 PM
67	Consultant apt was late contacting me then proceeded to talk over me stating he doesn't have time. If he permitted time for me to state any issues it would be better	1/29/2021 4:41 PM
68	To have been told in advance what time it would be at, instead of an unscheduled call. Working from home, had to excuse myself from meetings to take call, flustered and unprepared and forgot things I wanted to say.	1/29/2021 4:23 PM
69	I think face to face is much easier but given the current pandemic, it’s a good alternative.	1/29/2021 3:50 PM
70	It was very good, certainly better for a quick check in to make sure there aren't any problems.	1/29/2021 3:25 PM
71	It was very satisfactory indeed……no need to change	1/29/2021 3:09 PM
72	Just means can’t examin your joints . But better than no appointment	1/29/2021 2:56 PM
73	Visual	1/29/2021 2:39 PM
74	Not sure	1/29/2021 2:36 PM
75	Uncertain	1/29/2021 2:34 PM
76	I was pleased with having an appt this way the only downfall if you have a problem they cant examine your joints	1/29/2021 2:32 PM
77	Video call would have been better.	1/29/2021 2:31 PM
78	Possibly a pre appointment letter with tick boxes for points to be covered that would be done in a face to face appointment so that you can have all items to hand (such as BP readings, blood test results, Bath pain scores, photos etc	1/29/2021 2:30 PM
79	I would prefer a non face-to-face appointment to be conducted by a member of staff who knew me. I had a new doctor, so as well as losing that sense of personally caring, I felt a slight lack of continuity, although quality of care was as good. It just felt two steps removed from the personal touch.	1/29/2021 2:28 PM
80	Have a bit more time	1/29/2021 2:23 PM
81	It would have been better if the consultants had read the notes I'd sent to them before the meeting. As it was I had to go over everything again as they weren't prepared, and this wasted time and prevented a better question/answer response.	1/29/2021 2:14 PM
82	Follow-up sooner	1/29/2021 2:10 PM
83	No, very happy with telemedicine appointment.	1/29/2021 2:09 PM
84	Nothing comes to mind, it was functional.	1/29/2021 2:04 PM
85	Video would be preferable	1/29/2021 2:03 PM
86	I would have appreciated a video call, though I'm not really sure waving my swollen joints at a screen would have helped! It's just nice to see the person you're chatting to especially as one of my consultations was with a doctor I had not met before.	1/29/2021 6:55 AM
87	More time (with rheumatology) for thorough review; follow-up on some ongoing issues; usual blood tests. Video would have been preferred over telephone. GP and physio appts were perfect.	1/28/2021 11:49 PM
88	A follow up on if I had actually received the prescription & how it was helping - the said prescription never reached my gp & after various phone calls back to the hospital & my gp I gave up & suffered until I had an in person appointment	1/27/2021 6:09 PM
89	No worked better than expected	1/27/2021 3:31 PM
90	no	1/27/2021 2:48 PM
91	Nope	1/25/2021 9:38 PM

Q11 Do you feel it would be of benefit to you to be offered a telemedicine appointment as an option going forward (post Covid-19 pandemic)?

Answered: 107 Skipped: 22

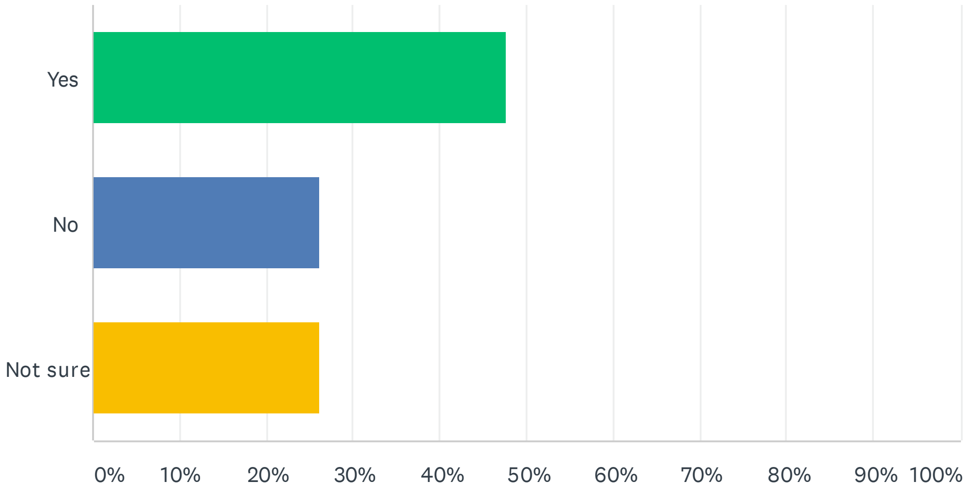

ANSWER CHOICES	RESPONSES	
Yes	47.66%	51
No	26.17%	28
Not sure	26.17%	28
TOTAL		107

#	PLEASE COMMENT:	DATE
1	Waiting for your views to go before taking vaccinations	3/9/2021 8:12 AM
2	AS is such a diverse disease symptom wise and sometimes physically seeing the doctor is more helpful. After I hung up I remembered several things I forgot to tell him.	3/1/2021 3:25 PM
3	I feel a 50/50 split between face to face and telemedicine is a pathway forward and it’s less visits to hospital.	2/27/2021 10:21 PM
4	but only if I don't have any particular problems, flares or new joints affected.	2/27/2021 4:52 PM
5	I have not had a dermatology consultation for over 3 yrs due to staff shortages and Covid. I would value a telemedicine appt.	2/24/2021 10:49 AM
6	In person appointments seem less rushed, more personal and allow Rheumatologist to examine joints.	2/16/2021 11:37 AM
7	I think it’s important that the consultant can see you at times when your symptoms are bad, but if things are settled then may be a telephone call is enough.	2/15/2021 10:58 PM
8	in times of pandemic … it will help the patients	2/15/2021 5:14 PM
9	It’s much better in person as sometimes it’s hard to discribe symptoms and you real need to be examined, also many treatments are not available over phone such as injections	2/12/2021 10:29 PM
10	A hybrid of telemedicine and in person would be good. Telemedicine for monitoring appointments	2/7/2021 3:03 PM
11	Already done with my Dermatologist: In my Phone review appointment last week, we discussed my psoriasis & psoriatic arthritis. Arranged for me to go in for a mole check in July. My Derm & Rheum. consultants work together because the psoriatic arthritis affects my skin & joints to varying degrees at different times	2/5/2021 7:44 AM
12	It is more comfortable to sit at home especially when the weather is bad. Also I am more relaxed and prepared.	2/4/2021 6:34 PM
13	Saves time, cost of travel etc. And if by video call is almost as good as face to face. Would be good for every other appointment.	2/4/2021 4:50 PM
14	Hugh saving on travelling and waiting offset by total lack of physical interventions/ monitoring. Probably ticks a few waiting list boxes but all in all absolutely no progress on managing my deteriorating condition.	2/4/2021 4:24 PM
15	Definitely not - just a waste of time. The patient's condition gets worse during the time waiting for a proper physical examination.	2/4/2021 3:16 PM
16	Face to face personal consultation and meetings are much better to talk openly, describe things, show things etc. The phone is ok for a brief question and answer, but not an annual or six monthly review.	2/4/2021 3:10 PM
17	I have both PsA and osteoarthritis. Without seeing someone I can’t identify what is causing pain.	2/4/2021 3:03 PM
18	I live over 100 miles away from my rheumatology unit, it is exhausting and difficult to attend appointments there. It would be very beneficial to have telemedicine appointments for most routine appointments.	2/4/2021 2:52 PM
19	In most situations if face to face follow possible if necessary	2/4/2021 2:49 PM
20	I travel over 2 hours to see one of my Rheumatologists so having a telemedecine appointment would be better.	2/4/2021 2:37 PM
21	I think it’s better to be seen in person at least once per year. There are subtle nuances to a person’s condition which cannot be detected by a phone consultation	2/4/2021 2:30 PM
22	It works but no contest with a face to face	2/4/2021 2:18 PM
23	Depending on situations of course, if someone is one remission for years telemedecine is a good option. if someone is under chronic pain, a physical appointment with a medical doctor can be key (provided that the medical doctor actually care about the patient, but that's another story...)	2/4/2021 2:16 PM
24	Yes, if it saves time for everyone and there isn’t a lot to say except ‘carry on’...... or would the practitioner miss something. At one visit in person, it was noticed that I had pneumonia and I wasn’t going to mention it!	2/4/2021 1:56 PM

25	It depends on what issues I have, some are more suited to telemedicine than others	2/4/2021 1:02 PM
26	It could be good if it were to be a video call - but i think it should be left quite fluid as in, it's booked as a video call BUT you can all the day before/week before to change to real life appointment if you need xrays etc. I know that the last appointment in Jan 2020 they wanted to get xrays redone of hands/feet/knees to see how bad the damage is, but obviously not had these since as all been phone appointments.	2/4/2021 12:45 PM
27	I would not mind, but would probably appreciate a face to face appointment once a year.	2/3/2021 2:06 PM
28	Only if my PSA is manageable and no concerns	2/3/2021 2:06 PM
29	I have to travel 100 mile round trip to the hospital, I’ve had more help and actually been listened to far more over the phone than 3 minutes face 2 face appointments	2/3/2021 12:56 PM
30	But would like a face to face at least once a year for joint assessment	2/3/2021 12:13 PM
31	Not always easy to determine oneself if everything is stable	2/2/2021 9:36 AM
32	S long as video rather than just telephone	2/1/2021 9:54 PM
33	Being seen and assessed face to face is too important with Psoriatic arthritis	2/1/2021 1:41 PM
34	Face to face will always be my preference but if telephone can help more urgently I wouldn't discount it	2/1/2021 12:53 PM
35	Useful for some follow up appointments, reduces travel and walking time. However it's ipmportant to continue face to face where physical examination would be helpful	1/31/2021 11:06 AM
36	There are times when face to face is necessary, and also it gives the doctor/nurse a chance to see the overall condition/appearance/mood of the patient. At othwer times, telemedicine could be helpful for "in- between" appointments	1/30/2021 6:24 PM
37	Less travel time for me and more efficient use of my time. I don't have to waste time waiting for apt in hospital.	1/29/2021 10:29 PM
38	f2f appointments offer the chance for the Doctor/nurse to see the joints, feel any swelling etc This can not be achieved over the phone	1/29/2021 8:12 PM
39	I feel my condition is better monitored by a proper physical examination by either a qualified nurse or by the consultant. It is difficult to put across joint problems by talking about them.	1/29/2021 8:01 PM
40	Provided that a face to face appointment would be available if I felt it was necessary	1/29/2021 7:55 PM
41	If there are no serious concerns it makes sense to have a telephone call or even as a triage call, it saves time for everyone.	1/29/2021 5:45 PM
42	It’s the future get on with it 😊	1/29/2021 5:44 PM
43	much easier than visiting hospital	1/29/2021 5:11 PM
44	Need some face to face to enable proper assessment	1/29/2021 4:42 PM
45	As long as it was scheduled in advance, maybe via zoom or MS teams etc	1/29/2021 4:25 PM
46	I can see the benefits but I think it’s harder to get information across. I’ve been having video calls with my physio but I had a face to face with her in clinic because she couldn’t understand why there was no improvement. She witnessed my knee giving way that couldn’t be seen in the video call.	1/29/2021 3:54 PM
47	As long as time is spent with the patient and not rushed. All usual questions asked and probed deeper than "any changes?"	1/29/2021 3:15 PM
48	Telephone call is fine but sometimes I feel important to also have option of face to face appointment	1/29/2021 3:15 PM
49	Would occasionally like a phone call but it shouldn't totally replace face to face contact.	1/29/2021 2:49 PM
50	I feel it should be a combination of both. Clearly it will save time to continue with phone appointments, but I do feel there are times when a visual is required as my GP would not have the same level of specific knowledge or expertise. Regarding Q12, (next question) it is not as conducive to openness with a doctor I have never met.	1/29/2021 2:43 PM
51	Attending the hospital for in person rheumatological appointment involves me taking 3 separate buses and could take over 6 hours for a round trip pre Covid. With Covid the frequency of buses has been reduced so would probably take even longer, or potentially involve an overnight hotel stay, all increasing the risks involved of catching Covid.	1/29/2021 2:43 PM

52	I am only partially diagnosed with PsA. I need a PsARC exam and an ultrasound for a firm diagnosis. (Despite NICE guidelines PsARCs are not routinely conducted. This is a widespread problem!) These exams cannot be done without face-to-face meetings. However, once diagnosed, I feel telemedicine appointments are very useful.	1/29/2021 2:38 PM
53	Telemedicine appointment is a great option for routine follow up appointments and certainly cuts out travelling time and making parking arrangements. However, if there are visible problems ie joint swelling, a description over the phone is nowhere near as helpful as in a clinic room setting where the Consultant is able to see you & do a physical examination. Making judgements on changing medications based only on telephone description can’t be as beneficial.	1/29/2021 2:35 PM
54	I think this way is good if your condition is under control	1/29/2021 2:34 PM
55	Visual contact better but face to face the best for diagnosis	1/29/2021 2:08 PM
56	Only occasionally	1/29/2021 2:05 PM
57	I would still like to see a medical professional once a year but not having to travel to Bath was a bonus.	1/29/2021 7:02 AM
58	Not adequate for regular six-month review, but if could have telephone appointments more or less when needed and space out face-to-face appointments longer than six months that would be more useful than rigid six-month face-to-face review.	1/28/2021 11:56 PM

Q12 Where do you feel you could be most open about your PsA when taking with your Rheumatologist?

Answered: 107 Skipped: 22

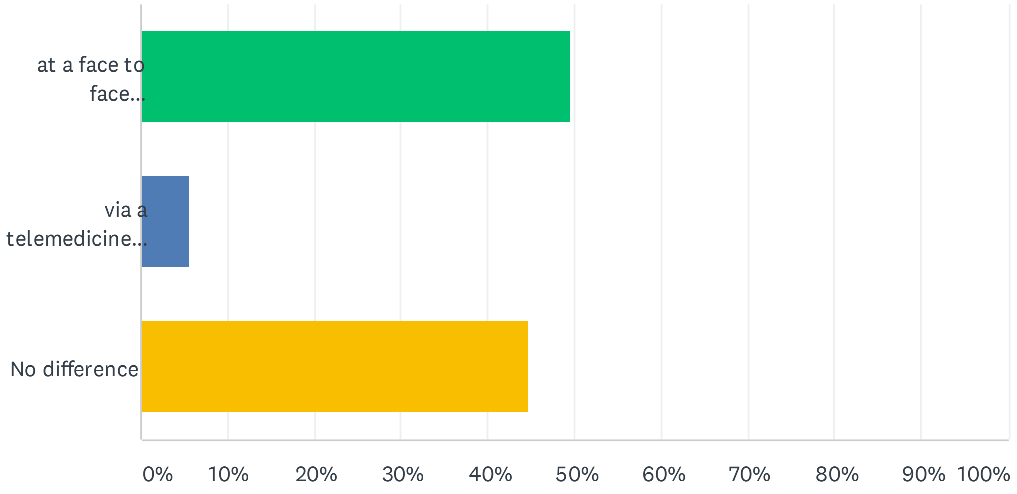

ANSWER CHOICES	RESPONSES	
at a face to face appointment	49.53%	53
via a telemedicine appointment	5.61%	6
No difference	44.86%	48
TOTAL		107

Q13 If your Psoriatic Arthritis symptoms are generally under control would you be happy to have your appointment conducted by either a Consultant or a suitably experienced rheumatology nurse?

Answered: 108 Skipped: 21

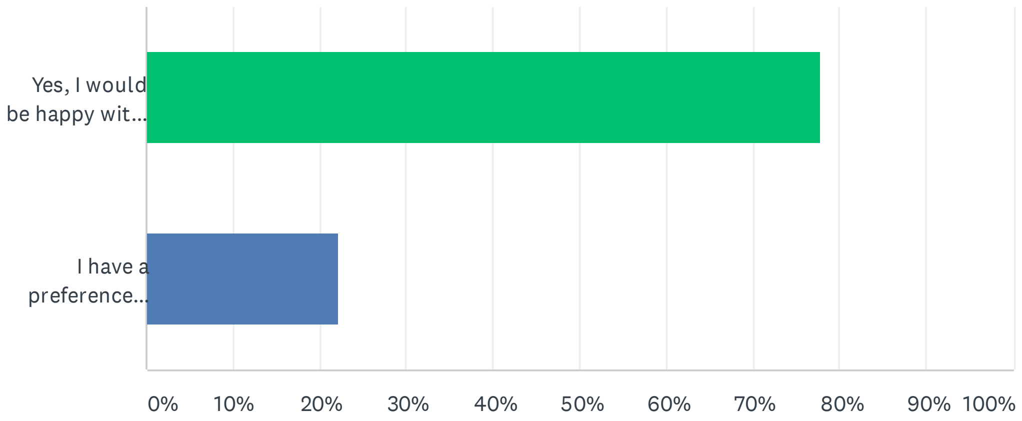

ANSWER CHOICES	RESPONSES	
Yes, I would be happy with either	77.78%	84
I have a preference (please tell us which and why):	22.22%	24
TOTAL		108

#	I HAVE A PREFERENCE (PLEASE TELL US WHICH AND WHY):	DATE
1	With expert only	3/9/2021 8:12 AM
2	Yes, but only if during that consultation it was decided that a physical attendance appointment was necessary, it was to be a as soon as possible and not have to wait for months..difficult I know.	3/1/2021 3:25 PM
3	I prefer to see the nurses, they are much more sympathetic to your problems, I think specialists see some horrible cases and forget some peoples symptoms may not be as server but they are still absolutely devastating to their lives. They also never explain things in layman’s terms	2/12/2021 10:29 PM
4	As long as it was fully under control and if it wasn't to be able to speak to a consultant, currently its like finding a needle in a haystack to speak to one	2/6/2021 11:15 PM
5	Consultant - I sometimes find others dismissive. They don’t know/understand the long and tortuous journey I went on before diagnosis.	2/5/2021 9:31 AM
6	My consultants, both of whom I have been seeing for >20 years, so they know me very well	2/5/2021 7:44 AM
7	A consultant has more discretion and power to effect treatments than a nurse.	2/4/2021 4:24 PM
8	Rheumatologist he finds stuff on palpation that I didn’t realise was a problem.	2/4/2021 3:27 PM
9	Consultant only - an experienced nurse could also be in the room at the same time.	2/4/2021 3:16 PM
10	Consultant who knows me and my history, specialist nurses often aren’t as up to date or knowledgeable as a Consultant in my past experience, and more likely to follow a script, whereas a Consultant can make a better judgement if treatment or testing needs to be changed.	2/4/2021 3:10 PM
11	Have never met a rheumatology nurse	2/4/2021 3:04 PM
12	I would insist on a consultant	2/4/2021 2:59 PM
13	I'd rather speak to the consultant as he can make changes more than a nurse can.	2/4/2021 2:37 PM
14	I prefer the specialist, as I feel that the nurse is following a script. If it is a telephone appointment, then surely the doctor can fit more patients in anyway?	2/4/2021 1:56 PM
15	I prefer a consultant because I have found they are happy to support me with a bespoke plan to manage my condition . When I have dealt with the lovely nurses they are completely text book and that hasn’t worked and in fact has put me into trouble	2/4/2021 12:00 PM
16	I only have one annual appointment with my rheumatologist and would not want this swapped to being with a nurse, although I’m happy to have nurse appointments inbetween the rheumatologist ones.	2/3/2021 1:13 PM
17	Consultant because I find the nurse often has to defer to the consultant as I’m quite complex	1/31/2021 1:36 PM
18	I would prefer to see a consultant and then have the nurse for me to email with any queries	1/31/2021 11:06 AM
19	I do value the qualified psoriatic nurse, but would also like to see my consultant occasionally if possible.	1/29/2021 2:43 PM
20	Pre Covid I usually had three monthly appointments, I.e. four a year, three of which would be with a nurse and one with a Consultant. I was very happy with this and would be happy to continue with it.	1/29/2021 2:43 PM
21	Consultant	1/29/2021 2:41 PM
22	Having not yet reached a stage where my PsA symptoms are generally under control, I would have concerns about attending appointments with a Rheumatology Nurse as the level of medical knowledge is vastly different to that of a Consultant or Registrar and I wouldn’t be confident. That said, if I did get to a point where symptoms and treatments were ok then I would have no problem with a Rheumatology Nurse appointment/ telephone call, more as a catch up / check in but not with the expectation of changing medication or treatment plan.	1/29/2021 2:35 PM
23	Prefer consultant as I have other issues	1/29/2021 2:24 PM
24	Would prefer consultant as early on in my treatment and things aren't yet stable even when often well. After a year or so of being stable I would happily switch to nurse by default and only see consultant if needed change in treatment.	1/28/2021 11:56 PM

Q14 How likely do you think ongoing remote consultations could impact your ability to achieve very good control (remission) of your condition?

Answered: 106 Skipped: 23

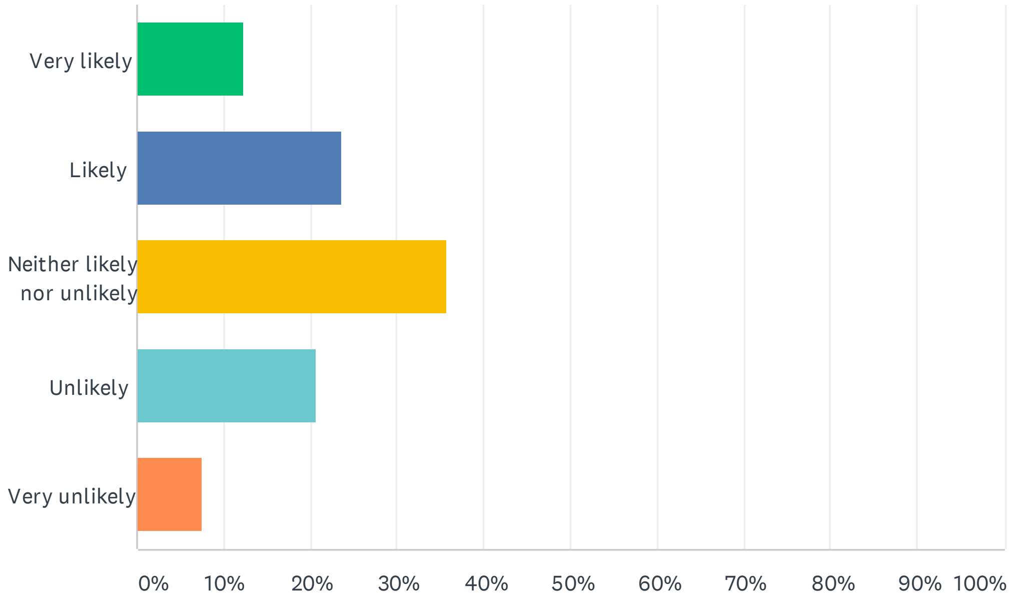

ANSWER CHOICES	RESPONSES	
Very likely	12.26%	13
Likely	23.58%	25
Neither likely nor unlikely	35.85%	38
Unlikely	20.75%	22
Very unlikely	7.55%	8
TOTAL		106

#	PLEASE ADD ANY COMMENTS:	DATE
1	It is hard to answer as the disease and problems fluctuate daily and may require you to be seen before your next scheduled telephone consultation.	3/1/2021 3:25 PM
2	After a telephone call last week, and concerns raised that medication is not as effective, specialist nurse arranged a face to face visit-in 5 weeks time to follow up	2/27/2021 10:21 PM
3	Really need face to face as not under control drugs need changing	2/19/2021 8:07 PM
4	Without proper examination of symptoms it’s impossible to monitor improvement or worsening of the conditions	2/12/2021 10:29 PM
5	Just increase the number of trainee consultants - don't bother with teleconsultations which hide the issue of not enough staff in the NHS. It also hides the number of those suffering - often in silence waiting, and waiting.	2/4/2021 3:16 PM
6	Last contact with secretary of rheumatologist got an appt for 10 months later	2/4/2021 3:04 PM
7	I think people should be seen in person, at least, once per year	2/4/2021 2:30 PM
8	I think as long as i feel that things are under control, it would save time for both parties for me to have a call, but again i think a video call would be more effective for this type of condition. Unless i really was having a good 6months.	2/4/2021 12:45 PM
9	My PA is not going to disappear so the consultations should be ongoing	2/4/2021 12:26 PM
10	This question is badly worded and confusing	2/4/2021 12:18 PM
11	The next step for me would be biologics which I don't currently qualify for. The only way to qualify is to be examined for swollen/tender joints. I was called in for a face to face as a consequence of my phone consult though so I feel it met my needs	2/3/2021 4:26 PM
12	It’s difficult to express the pain when having to describe where it is rather than showing exactly where....	2/3/2021 2:06 PM
13	I find this question very difficult to understand, so I’m not sure that I’ve answered it correctly. My situation is that I’m currently under fairly good control so telemedicine appts ok for me right now, but if I began to flare/needed new meds then I imagine that no face to face appointments might negatively affect me switching meds quickly.	2/3/2021 1:13 PM
14	Because I find on the phone the appointment is more in depth	2/3/2021 12:56 PM
15	Very useful to not have to go if stable, but then face to face when needed, physical specialist review	2/1/2021 9:54 PM
16	I cannot be assessed for a change in medication unless I am seen face to face. I am poorly controlled	2/1/2021 1:41 PM
17	So long as access to specialist is available when needed, when one is in a non-active phase, a check-up from a suitably qualified nurse is fine	2/1/2021 12:53 PM
18	.	1/31/2021 9:46 PM
19	I think face to face is important at least annually to carry out a physical assessment of specific symptoms and joint issues	1/31/2021 1:36 PM
20	Freeing up more time for consultant means more apts available, meaning more catch ups.	1/29/2021 10:29 PM
21	Please see previous comment.	1/29/2021 8:01 PM
22	Hard to say as it is not all that long since I was diagnosed and my pain is currently not under control	1/29/2021 7:55 PM
23	I think control of my condition will be fine. This question is confusing.	1/29/2021 5:45 PM
24	It isn’t possible to see the joints and inflammation, and level of movement over the phone	1/29/2021 5:07 PM
25	My condition is well managed and under control, thank you all at “The New Min”. My right hip joint is just a little suspect occasionally	1/29/2021 3:15 PM
26	It depends how long this goes on for. I am not always so open on the phone.	1/29/2021 2:49 PM
27	This question is badly worded and difficult to answer as it's clear meaning is difficult to unpick. I think patients need to be seen occasionally as aspects of the disease are visual.	1/29/2021 2:43 PM
28	Because of the seronegative and the very poorly understood nature of PsA, by many rheumatologists and related medical staff, I feel that neither remote nor face-to-face	1/29/2021 2:38 PM

	consultations will help me achieve control of my condition.	
29	With sufficient time allowed to discuss and with an already established good relationship with my Consultant, I don’t doubt that I will still achieve remission of my condition despite my appointments being carried out remotely.	1/29/2021 2:35 PM
30	BUT depends on appearance of unlocked for developments	1/29/2021 2:08 PM
31	I have never really been in remission and after thirty years I don't think a telephone appointment will effect that!	1/29/2021 7:02 AM

Q15 How likely do you think ongoing remote consultations could impact your ability to take part in clinical research studies?

Answered: 105 Skipped: 24

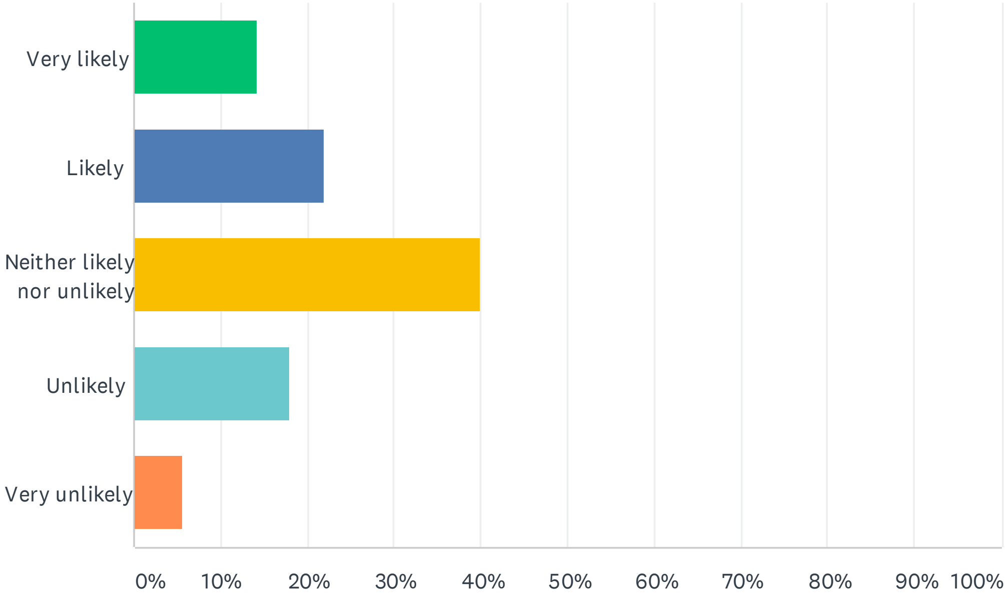

ANSWER CHOICES	RESPONSES	
Very likely	14.29%	15
Likely	21.90%	23
Neither likely nor unlikely	40.00%	42
Unlikely	18.10%	19
Very unlikely	5.71%	6
TOTAL		105

#	PLEASE ADD ANY COMMENTS:	DATE
1	I've never been asked to take part in a clinical research study, so I don't know if it would make a difference	2/27/2021 4:52 PM
2	I’d like to take part in a study but have not been told of any	2/12/2021 10:29 PM
3	I’ve never been asked to take part in any clinical research studies. It seems to me that most research is concentrated on RA	2/4/2021 2:30 PM
4	People tends to underestimate their state - i think doctors should witness the evolution before a decision is made	2/4/2021 2:16 PM
5	This question is also badly worded and confusing	2/4/2021 12:18 PM
6	I’d like to take part and not having to travel makes a huge difference All my bloods/x rays are done at a more local hospital 40 mile round trip but all rheumy appointments are 100 mile round trip so over the phone is great	2/3/2021 12:56 PM
7	These can still be offered remotely, with online questionnaires, interactions and remote video interviews, blood boxes sent and GP can take bloods locally and post	2/1/2021 9:54 PM
8	They mostly recruit by letter having gone through your medical notes anyway	2/1/2021 1:41 PM
9	More catch ups means increased contact time so more chances of being invited for clinical trials	1/29/2021 10:29 PM
10	I would expect any trials to be designed with this point in mind	1/29/2021 7:55 PM
11	Start asap	1/29/2021 5:44 PM
12	I worry about vital information being missed in the video calls	1/29/2021 3:54 PM
13	The research studies I have taken part in tend to be form filling based. That could be done online with appropriate safeguards.	1/29/2021 2:49 PM
14	They seem to have disappeared, but then everyone is busy with the pandemic anyway at this time.	1/29/2021 2:43 PM
15	I take part in ongoing research projects that require me to give an extra blood sample when attend ing the hospital.	1/29/2021 2:43 PM
16	Depending on the nature of the study I feel they could mostly assist with my ability of take part in with clinical research studies.	1/29/2021 2:38 PM
17	I am still very happy to take part in anything that might help, regardless of whether this is carried out remotely.	1/29/2021 2:35 PM
18	During lockdown I have signed up for three (not rheumatology) studies, if anything it has been made easier by the use of online resources.	1/29/2021 7:02 AM

Q16 Do you think ongoing remote consultations would be able to help provide as good, well-rounded ('holistic') care as regular face-to-face appointments with the clinical team?

Answered: 105 Skipped: 24

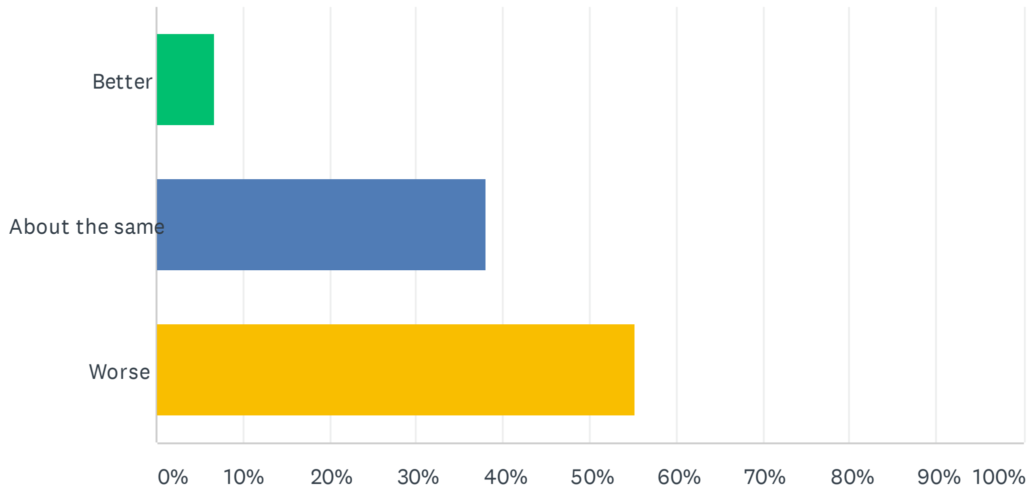

ANSWER CHOICES	RESPONSES	
Better	6.67%	7
About the same	38.10%	40
Worse	55.24%	58
TOTAL		105

#	PLEASE COMMENT (OPTIONAL)	DATE
1	Yes, if the remote consultations decreases the time between clinic appointments, thus more frequent check-ups	2/27/2021 10:21 PM
2	I think they would be better (more convenient) for me than a face-to-face appointment, but if I was having problems and my joints were getting worse, I would definitely prefer to have a face-to-face appointment, esp. if they wanted to increase my dose of methotrexate.	2/27/2021 4:52 PM
3	However non-verbal communication is obscured with just an aural channel. It might be harder to judge how I feel about the answers I am giving. I might be prepared to answer if asked directly.	2/27/2021 9:14 AM
4	Easier to organise, less time pressure.	2/24/2021 10:49 AM
5	I'd worry if all my appointments became telephone calls .. I do think the consultant could miss things if they didn't actually see you.	2/15/2021 10:58 PM
6	Perhaps have every 2nd or 3rd visit face to face for an examination. The consultant could see some problem that I don't know about, eg skin melanomas or other problems. I have an open appointment at Harrogate hospital anyway, so I can call the departments if I have any skin or joint flare-ups between review appointments.	2/5/2021 7:44 AM
7	Very good for tick box driven regime but in terms of managing my condition it's been useless. Under current conditions with all non Covid-19 treatments stopped its just a useless placebo as there is no physical intervention available. I'm left with a feeling of gradually slipping off the radar. Abandonment no less.	2/4/2021 4:24 PM
8	You pay good money and wait for a long time to see a rheumatologist. The physical examination of joints movements etc effects management/ treatment.	2/4/2021 3:27 PM
9	You can't beat the person to person interaction. The personal touch is much better.	2/4/2021 3:10 PM
10	See previous comments. It's much easier to be " fobbed off " on the phone	2/4/2021 2:30 PM
11	Face to face appointments are far to be holistic. You are just another case to whom the answer is either to give more or less drugs	2/4/2021 2:16 PM
12	Face to face discussions are. Very often more useful as for example, something that the doctor does triggers further discussion	2/4/2021 1:02 PM
13	It is always good to have some face to face interaction with your healthcare provider. I know that mine feels all my joints and is able to pick up on swelling that I don't really notice myself, so me saying "no swollen joints" could be false information as I am not trained medically like she is. Plus you build a better rapport with someone face to face. I am lucky and have been with mine since 2006 so we know each other very well. But for new diagnoses it is very important for the face to face care to happen.	2/4/2021 12:45 PM
14	Sometimes it is better for the consultant to see the problem - perhaps a zoom consultation could achieve this?	2/4/2021 12:26 PM
15	I think you would need to see a consultant when there is a change /problem with your condition	2/4/2021 12:04 PM
16	Depends on what my concerns are at the time. There will be occasions when face to face will be better as stated previously	2/3/2021 4:26 PM
17	I think it would be fine for 'stable' patients but face to face appointments need to be available for people whose symptoms are not stable ... at the very least they need to be video rather than just audio	2/3/2021 1:13 PM
18	I think the holistic care would be lost, but as an out of area patient I currently do not get this part of the care. Bath nurse specialists are amazing always offer help and support when I needed it.	2/1/2021 9:54 PM
19	As pointed out before Rheumatology is too hands on	2/1/2021 1:41 PM
20	You can't see inflammation and swollen joints, or lack of movement over the phone	2/1/2021 1:05 PM
21	Much depends on the ability of the patient to express themselves, and, of course, the ability of the doctor or nurse to understand, translate and express themselves clearly. Vocal inflexion is only a small but significant part of human interaction. The question of languages in a multi-racial community is important.	2/1/2021 12:53 PM
22	I think both are necessary and valuable	1/31/2021 1:36 PM

23	They might be useful as part of a mix but I don't think.it would be ideal to only ever have remote consultations	1/29/2021 7:55 PM
24	No travel stress ,zoom for face to face , just need to work out none zoom folk friends relations	1/29/2021 5:44 PM
25	Need some face to face	1/29/2021 4:42 PM
26	I am more open in face to face.	1/29/2021 2:49 PM
27	1. The momentum of follow-through is lost - blood tests, x-rays, visual examination, assessment of joint pain and swelling. 2. There is also the feeling of being somewhat 'parked' - 'holisitc' care is also about how the patient feels cared for.	1/29/2021 2:43 PM
28	I think the more that is learned about conducting effective remote consultations the better they will become. There are negative and positive elements, in particular to non-verbal communications, e.g. landline phone calls. Also, my experience of video call consultations has been mixed, one was chaotic, the other was more streamlined. Effective protocols for different types of communication need to be developed and followed to achieve the best results for all parties involved.	1/29/2021 2:38 PM
29	Would be okay for a limited period but face to face would be needed at some point to check out body conditions	1/29/2021 2:13 PM
30	Without being able to see the patient it is difficult to tell if they are holding something back that could be teased out in the consultation, especially around them suggesting things for themselves.	1/29/2021 7:02 AM
31	Depends if they completely replaced previous face to face or were used in conjunction with.	1/28/2021 11:56 PM

Q17 Do you think ongoing remote consultations will allow you to build as good a relationship / rapport with your clinical team as regular face-to-face appointments have done to date?

Answered: 105 Skipped: 24

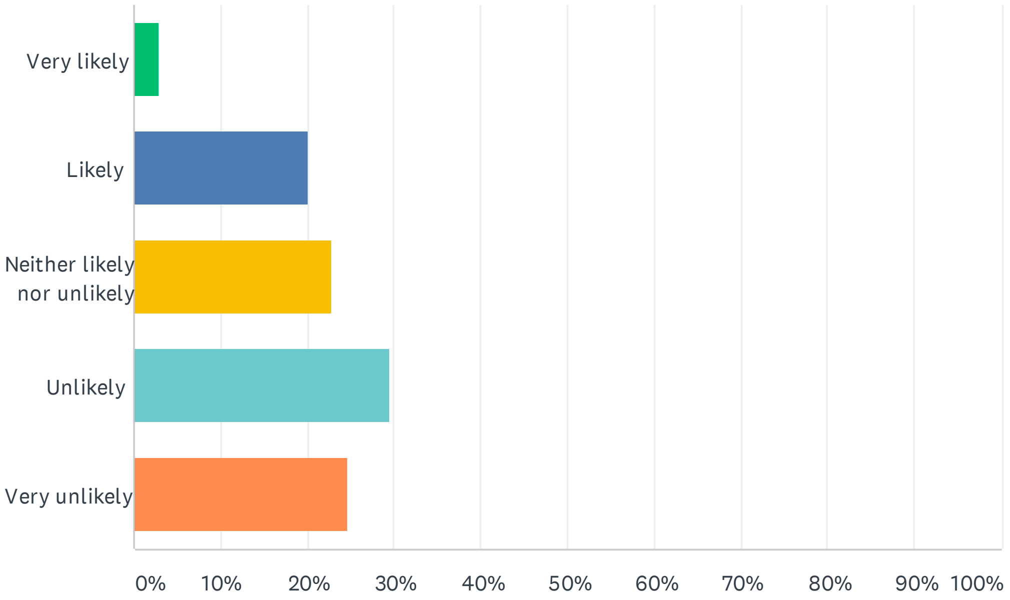

ANSWER CHOICES	RESPONSES	
Very likely	2.86%	3
Likely	20.00%	21
Neither likely nor unlikely	22.86%	24
Unlikely	29.52%	31
Very unlikely	24.76%	26
TOTAL		105

#	PLEASE COMMENT (OPTIONAL)	DATE
1	I do feel 1st appointments face to face with consultant and specialist nurse is vital. Also, if patient medication is no longer managing symptoms	2/27/2021 10:21 PM
2	New way of working but I can adapt and I'm sure professionals can as well.	2/24/2021 10:49 AM
3	It’s really hard to have an in-depth conversation or build a real relationship over the phone	2/12/2021 10:29 PM
4	I suspect all calls are recorded and monitored so don’t expect an honest interaction	2/4/2021 4:24 PM
5	Its all about saving money now what about the human interactions . There is a place for telemedicine but rheumatology is all about feel and movement of joints the camera cannot do that .	2/4/2021 3:27 PM
6	A faceless voice on the phone can’t possibly build a relationship between patients and consultants without meeting in person.	2/4/2021 3:10 PM
7	I never seem to see the same staff on face to face appointments anyway.	2/4/2021 2:52 PM
8	As above - no. Face to face is really needed to get to know someone and build that rapport and good relationship.	2/4/2021 12:45 PM
9	Only time would tell -zoom meeting style consultations may be better	2/4/2021 12:26 PM
10	I do think for new patients this will be very difficult going forward, but intermittent face to face will hopefully help with this	2/1/2021 9:54 PM
11	It would be difficult to develop as close a relationship as I have with the existing team if a new member of staff came on board who didn’t know me from Adam	2/1/2021 1:41 PM
12	For the reasons I have given above. Face-to-face diagnosis is difficult enough and usually needs tests to validate diagnosis, by voice and language only adds many complications	2/1/2021 12:53 PM
13	I feel my direct contact with the team has built up valuable trust and understanding of my difficulties as a patient.	1/29/2021 8:01 PM
14	This very much depends on the individuals (patient and clinician) involved	1/29/2021 7:55 PM
15	There is no reason face to face cannot be done over phones iPads etc	1/29/2021 5:44 PM
16	I prefer to see the person I am talking to.	1/29/2021 2:49 PM
17	How could that be possible, especially with new team members we have never met?	1/29/2021 2:43 PM
18	I am fortunate to have met with and built a good rapport with my consultant prior to the COVID restrictions so remote consultations are easier to manage. It must be very difficult to have not had this opportunity and to be coping with telephone appointments having never met the Clinician you are talking to.	1/29/2021 2:35 PM
19	Part of building those relationships is about the conversation before and after the technical part of the conversations, the what have you been up to and having a laugh about things. This is the thing I also miss most about working from home and not seeing my work colleagues.	1/29/2021 7:02 AM
20	Do you build up a rapport with people over the phone in the same way as people you meet face to face?	1/28/2021 11:56 PM
21	Consultant changes so often difficult to build relationships	1/27/2021 3:33 PM
22	Depends on whether you speak to someone you have met/knows your history. If it's a new person it might be difficult to build a good rapport?	1/27/2021 2:49 PM

Q18 Before you go, is there anything else you'd like to share about your experience of Telemedicine?

Answered: 32 Skipped: 97

#	RESPONSES	DATE
1	My experiment is as below 1. Not no eat Weat in any form . Must Eat Gluten free food . Never eat citrus and preservative food . Can use Leamon (ascorbic acid or Malic acid We can eat) Synthetic leamon , vinegar r worst . Gram , rice , bajra , Ragi is good to eat . More Experiment and experience I can share In next	3/9/2021 8:24 AM
2	no thanks	3/1/2021 3:25 PM
3	My appointment happened early - the day before my scheduled appointment - which was fine, although I didn't feel fully prepared for it. Nevertheless - it was still very useful and a big help that I could talk to my consultant without having to go to the hospital.	2/27/2021 4:54 PM
4	If a face to face followup if required after a telephone consultation this still is subject to delays during a pandemic	2/27/2021 9:16 AM
5	Saves everyone time and money!	2/5/2021 7:45 AM
6	It is a bit frivolous but it saves me spending money in Bath!	2/4/2021 6:41 PM
7	Just my disappointment at how few doctors have embraced video consultations.	2/4/2021 4:52 PM
8	It is a good option in very remote areas , where it is impossible to see a specialist face to face .	2/4/2021 3:28 PM
9	Makes me feel I’m unimportant, a faceless voice.	2/4/2021 3:11 PM
10	No	2/4/2021 2:59 PM
11	If you have a long or difficult journey to a rheumatology unit, telemedicine can be very useful.	2/4/2021 2:53 PM
12	I felt that the doctor was more relaxed over the phone. This is also to consider on top of the patient experience.	2/4/2021 2:17 PM
13	I think everyone is doing an amazing job considering the state of the world currently. The appointments I have had have been very good quality, but I do think a video/face to face is better for myself personally. However, I am more than happy with the level of care I have received during this pandemic.	2/4/2021 12:46 PM
14	It could be a saving capacity for NHS as consulting rooms would not be required saving ££ on electricity, heating, cleaning , wear and tear of equipment, furniture, fewer reception staff required. Also it could free up the nurses to go on wards	2/4/2021 12:30 PM
15	Not sure if older people will be okay with technology aspects.	2/4/2021 12:17 PM
16	No but they don't see what your joints are like through a phone	2/4/2021 12:05 PM
17	When I received a copy of my appointment outcome letter.... my flaring finger joints were described on the wrong side of my body...	2/3/2021 2:08 PM
18	Have had problems with another discipline giving wrong website details so failed to connect	2/3/2021 12:15 PM
19	No thank you	2/1/2021 3:07 PM
20	You need to be able to see someone so as not to lose the visual cues. I could put on a happy voice but it is hard to ‘act’ happy and pain free is you’re actually seen	2/1/2021 1:44 PM
21	I understand that telemedicine is likely to stay, and that it can help to make the service more efficient but I believe it to be only partially effective in diagnosis. However this needs significant research to prove or disprove.	2/1/2021 12:55 PM
22	no	1/30/2021 6:24 PM
23	It’s going to be phenomenal for other things like wearables,so the nhs should lead the world by hiring watches to the vulnerable ect	1/29/2021 5:48 PM
24	I think the telemedicine appointments could work very well combined with face-to-face appointments.	1/29/2021 5:46 PM
25	No	1/29/2021 2:49 PM
26	I much prefer it as I live in a rural community with long (30) mile trips to all of the major hospitals. I don’t drive and public transport is inadequate to them all. Taxis can cost up to £40 each way. Patient transport services are limited and I do not qualify. Telemedicine is something I have been championing for some time with both Arthritis charities and Age UK and I would have welcome an expansion of it.	1/29/2021 2:48 PM

27	I feel they should be something that suits patients of all backgrounds and in all socio-economic groups.	1/29/2021 2:41 PM
28	No	1/29/2021 2:25 PM
29	Voice is better than nothing but is no substitute for face to face when more subtle or unintentional signals may be detected	1/29/2021 2:11 PM
30	Much prefer face to face appointment	1/29/2021 2:06 PM
31	It didn’t work for me!	1/27/2021 6:11 PM
32	I think it's a very versatile option for both - and sometimes more useful when I wouldn't really have the energy to get out.	1/27/2021 2:50 PM
