# Supplementary material for: Patients’ perspectives of telemedicine appointments for psoriatic arthritis during the COVID-19 pandemic: results of a patient-driven pilot survey
Source: BMC Rheumatol. 2022 Feb 22;6:13. doi: 10.1186/s41927-021-00242-y (PMC8860501; doi:10.1186/s41927-021-00242-y)
Supplement: Supplementary file 2 — Additional file 2: Lay summary. [file 41927_2021_242_MOESM2_ESM.docx]

**Patients’ perspectives of telemedicine appointments for psoriatic arthritis during the COVID-19 pandemic: results of a patient-driven pilot survey – article summary**

Hannah Jethwa and Melanie Brooke

The outbreak of the COVID-19 pandemic triggered a dramatic shift in the way outpatient healthcare is delivered in the UK and it is likely that virtual consultations, via either or video-conference calls, will remain a standard aspect of clinical care delivery in the future. This article summarises the results of a patient-driven survey to review patients’ perspectives of telemedicine appointments relating to psoriatic arthritis. There has been a recent drive to increase patient involvement in research and there is a possibility that a survey designed and implemented ‘by patients for patients’ may allow for freer and more honest expression from respondents as well as have more focus on issues important to patients themselves, rather than those perceived to be significant to them from healthcare practitioners’ perspectives. From our survey five categories were identified which highlight patients’ views on their virtual consultations, including the necessity for physical assessments, the benefits of face-to-face assessments in those with physical symptoms, the requirement for a flexible and responsive system in case appointments need changing, the benefits of prior relationships with healthcare providers prior to virtual consultations and the acknowledgement that telemedicine is likely to continue in the long-run.
